# Size and Fluorescence Properties of Algal Photosynthetic Antenna Proteins Estimated by Microscopy

**DOI:** 10.3390/ijms23020778

**Published:** 2022-01-11

**Authors:** Aurélie Crepin, Erica Belgio, Barbora Šedivá, Eliška Kuthanová Trsková, Edel Cunill-Semanat, Radek Kaňa

**Affiliations:** 1 Centre Algatech, Institute of Microbiology of the Czech Academy of Sciences, Opatovický Mlýn, 379 81 Třeboň, Czech Republic; belgio@alga.cz (E.B.); sediva@alga.cz (B.Š.); kuthanova@alga.cz (E.K.T.); semanat@alga.cz (E.C.-S.); 2 Umeå Plant Science Centre (UPSC), Department of Plant Physiology, Umeå University, 901 87 Umeå, Sweden

**Keywords:** photosynthesis, antenna proteins, light-harvesting, fluorescence correlation spectroscopy, protein diffusion, microscopy, protein oligomerization, *Chromera velia*

## Abstract

Antenna proteins play a major role in the regulation of light-harvesting in photosynthesis. However, less is known about a possible link between their sizes (oligomerization state) and fluorescence intensity (number of photons emitted). Here, we used a microscopy-based method, Fluorescence Correlation Spectroscopy (FCS), to analyze different antenna proteins at the particle level. The direct comparison indicated that Chromera Light Harvesting (CLH) antenna particles (isolated from *Chromera velia*) behaved as the monomeric Light Harvesting Complex II (LHCII) (from higher plants), in terms of their radius (based on the diffusion time) and fluorescence yields. FCS data thus indicated a monomeric oligomerization state of algal CLH antenna (at our experimental conditions) that was later confirmed also by biochemical experiments. Additionally, our data provide a proof of concept that the FCS method is well suited to measure proteins sizes (oligomerization state) and fluorescence intensities (photon counts) of antenna proteins per single particle (monomers and oligomers). We proved that antenna monomers (CLH and LHCIIm) are more “quenched” than the corresponding trimers. The FCS measurement thus represents a useful experimental approach that allows studying the role of antenna oligomerization in the mechanism of photoprotection.

## 1. Introduction

Phototrophic organisms carry large, multi-protein complexes that absorb light and drive photosynthetic reactions leading to charge separation. They have to cope with a constantly changing quantity and quality of light. Several physiological responses are in place to maximize photosynthetic efficiency in the most heterogeneous conditions (see e.g., [1,2,3,4,5,6]). One key regulation point is placed at the level of photosynthetic antenna proteins. Bound to the core of photosystems, the antenna protein complexes bear the double function of light harvesting (useful for photochemistry) and excess energy dissipation participating in photoprotection. The way this molecular switch between the light-harvesting and photoprotective mode occurs is the object of many studies (reviewed for instance in [7,8]). One important factor, often undermined, is protein–protein interactions and in vivo oligomerization state of antenna complexes. It is important to note that antenna proteins in vivo can be found both in the form of monomers and multimers of various sizes [9,10,11,12]. This factor is not trivial as recent evidence indicates that, like for other proteins, it impacts also antenna protein function. Studies on intact chloroplasts and isolated light-harvesting complexes of higher plants (LHCII), for example, indicated that the trimeric LHCII complexes are exceptionally well adjusted to absorb light quanta and use them for photochemistry, while LHCII monomers are better in excess light energy dissipation [13,14]. A similar difference exists between LHCII trimers and native monomeric LHC antennas, with the second being considered as more efficient in energy dissipation [15]. Further, it has been suggested that light-driven changes affecting antenna oligomerization could take part in the regulation of energy dissipation in the thylakoid membranes [14,16,17]. Therefore, gathering information on antenna oligomerization state could help provide a better comprehension of not only protein function but also thylakoid membrane organization involved in the regulation of photosynthesis [18].

The oligomerization state of antenna proteins is, however, not always straightforward to determine. Protein crystallization, challenging by itself, is further complicated by the presence of detergent and chemicals which affect particle–particle interactions [19]. Gel electrophoresis and gel filtration provide useful information on protein sizes, but they require/assume similarities with known proteins or standards [6,20,21]. Finally, purified antenna proteins are often too small and round in shape to achieve sufficient high-resolution electron microscopy images. As a matter of fact, most of the structures solved by cryo-electron microscopy involve antennas bound to core complexes [9,10,11,12,22,23,24,25,26,27,28,29]. None of those methods, however, is able to establish simultaneously the oligomerization state of antenna proteins together with some other physiological measurements (e.g., chlorophyll a fluorescence measurements).

Concerning algae, the problem of antenna oligomerization is further complicated by larger structural variations compared to plants [10,11,12,20,30,31]. A good example of this protein complexity is represented by the CLH (Chromera Light-Harvesting) antennas from the apicomplexan alga *Chromera velia*. CLH complexes were shown to comprise protein bands between 18 and 19 KDa [21,32]. The apoproteins are estimated to bind eight chlorophylls (Chl) (from the conservation of binding sites [33]) and three to five carotenoids (Car) (from Chl:Car ratios [21,32]). The purified CLH antennas were reported to be present in several different oligomerization states within a single purified sample (e.g., [5,6,32]). The smallest particles were described either as trimers [5,32] or as monomers [6].

Fluorescence Correlation Spectroscopy (FCS) represents a quasi-single molecule method and a promising approach to answer these questions. It allows determining the oligomerization state of purified antenna proteins simultaneously with their fluorescence measured as a photon count per particle. We have recently used it to characterize changes in the large particles formed by plant Light Harvesting Complex II (LHCII) antennas, and their tendency to form clusters upon changes in detergent and pH conditions in vitro [34].

Here, FCS allowed us to study algal CLH protein particles as isolated from native membranes and compare their sizes and fluorescence intensities with the monomeric and trimeric antennas from higher plants (LHCII). We confirmed that FCS is sensitive enough to distinguish between antenna monomers and trimers. Further, the method showed that algal CLH antennas from *C. velia* are mainly present in the form of monomers in the conditions we tested. Additionally, the fluorescence yield of CLH particles was also close to that of the monomerized form of LHCII from plants. We discuss possible implications of the results and applicability to study the non-photochemical quenching processes considering protein sizes.

## 2. Results

### 2.1. FCS Points to a Monomeric State of CLH

**Figure 1:**
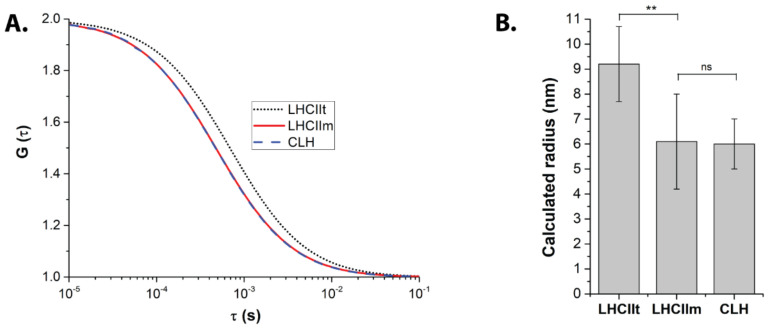
Oligomerization state of light-harvesting antennas estimated from autocorrelation correlation functions (ACFs) in microscopic Fluorescence Correlation Spectroscopy. Isolated antennas of trimeric LHCII (LHCIIt), monomerized LHCII (LHCIIm) and CLH antennas were measured in conditions of high pH (7.3) and high detergent concentration DDM (200 μM). (**A**) Averaged ACFs (G (τ)) for antenna proteins in solution. Data represent the results of the fitting. See Supplementary Figure S1 for raw traces and residuals. (**B**) Radius of the antenna particles as calculated from ACFs (see Materials and Methods). Data represent averages and standard deviations of 7 independent measurements. All samples were measured at the same OD_676._ A double asterisk marks very statistically different data groups; ns = not statistically different.

We used Fluorescence Correlation Spectroscopy (FCS) to investigate the oligomerization state of light-harvesting antennas purified from a higher plant (LHCII) and an alga (CLH). First, we assessed the FCS method’s resolution by measuring the monomeric and trimeric forms of the same protein, LHCII (LHCIIm and LHCIIt, respectively). The acquired auto-correlation functions (ACFs) (Figure 1A) changed significantly with LHCII monomerization. Indeed, the estimated average radius decreased from 9.2 ± 1.5 nm to 6.1 ± 1.9 nm in LHCIIt and LHCIIm, respectively (Figure 1B, Table 1). For the same chlorophyll concentration (see Materials and Methods), the overall number of particles detected in the confocal volume increased for the monomerized LHCII (Table 1). Both these results confirmed the ability of the FCS method to resolve the small size differences between the monomer and trimer of LHCII.

The method was then applied to the antenna proteins of unknown oligomerization state—purified CLH from *Chromera velia* (Figure 1, Table 1). Their acquired ACF curves showed a diffusion time comparable with LHCII monomers (see Table 1). The radius estimated for CLH was 6.0 ± 1.0 nm, almost identical to LHCIIm (Table 1 and Figure 1B). Also in this case the number of particles increased significantly compared to LHCIIt (Table 1). FCS data thus suggested that purified CLH proteins in our conditions display size close to the monomeric state of LHCIIm.

### 2.2. CLH and Monomeric LHC Proteins Co-Migrate on Sucrose Gradients

**Figure 2:**
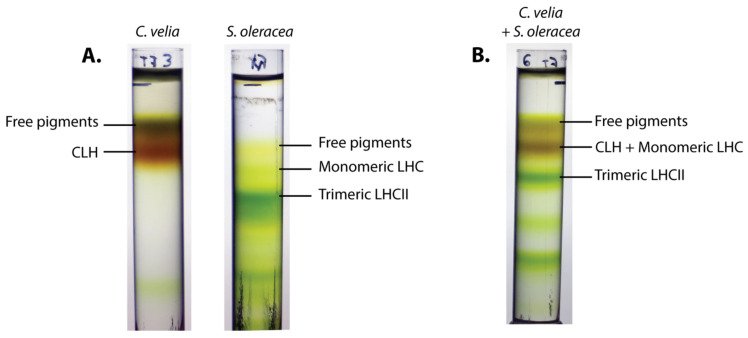
Separation of solubilized thylakoid membranes from *C. velia* or *S. oleracea* (spinach) on sucrose gradients. (**A**) Solubilized thylakoids from each organism were deposited on identical sucrose gradients and centrifuged in the same conditions. The band corresponding to CLH antenna from *C. velia* laid just below the free pigments one, at a position correspondent to monomeric LHC antennas from spinach. (**B**) Membranes from both organisms were deposited and centrifuged on a single gradient, resulting in an apparent co-migration of monomeric LHC and CLH.

FCS results were further validated by a biochemical method (Figure 2). The thylakoid membranes of *C. velia* and *S. oleracea* were solubilized using published protocols [34,35], and run in parallel on identical sucrose gradients (Figure 2A). The brown band in the membranes solubilized from *C. velia* corresponded to the typical band identified in several previous reports as CLH [5,21,32]. It was found immediately below the free pigment fraction, at the level of a band well described previously as monomeric LHC proteins in plants (Figure 2A) (see e.g., [36,37]). The characteristic band of trimeric LHCII, instead, was found lower in the plant gradient, without a counterpart in *C. velia* in these conditions (Figure 2A). When solubilized membranes from the two different organisms were mixed and loaded onto the same gradient, monomeric LHC proteins and CLH particles appeared indistinguishable: they migrated in a single band (Figure 2B), clearly separated from the LHCIIt. The biochemical data together with FCS analysis (Figure 1) thus indicated that CLH antennas (in the condition that we used for isolation) are characterized by a low oligomerization state, close to that of monomeric antennas.

### 2.3. Fluorescence Yields of Antenna Proteins Varied with Their Oligomeric States

Fluorescence intensity (i.e., photon Count per Protein Particle—CPP) was estimated in the purified antennas based on analysis of FCS data as the total fluorescence detected in the measurement (Total Photon Count Rate, see Table 2) divided by the number of antenna particles (Table 1). Considering LHCII, despite the three-times decrease in chlorophyll content per protein between LHCIIt and LHCIIm, the fluorescence emission per LHCIIm particle was diminished only by 30% compared to LHCIIt (see Table 2). The CPP of CLH was even lower than LHCIIm (see Table 2). However, when normalized to the expected number of chlorophylls per antenna particle, monomeric antennas (LHCIIm, CLH) displayed a similar fluorescence yield. On the contrary, these two values were very significantly higher than the one from LHCIIt (Table 2). Based on CPP normalized to chlorophyll content per protein, the trimeric LHCII antenna particles thus displayed the lowest fluorescence yield per pigment compared to monomeric antennas of CLH and LHCIIm, which were almost identical in the number of photons per single chlorophyll (see Table 2).

## 3. Discussion

Particle oligomerization is one of the factors affecting antenna protein function [13,14], including regulation of energy transfer and, in turn, photosynthetic efficiency [14,16,17,38]. Several methods provide information on protein size, from basic biochemical methods to crystallography or electron microscopy. These techniques, however, provide rather static information about protein size and organization in a particular experimental condition used for sample preparation. There is also need for simple approaches and workflows able to study the antenna particles dynamically, as a function of a changing environment (e.g., pH, ions, etc.). This is, however, an advantage of the FCS technique, which can study particles sizes in a wide range of conditions.

FCS is able to study protein interaction, diffusion, and photophysics directly in solution based on fluorescence fluctuations at the microscopic level [39]. Although to this day, the method has rarely been used in photosynthesis research, it proved to be well suited for the study of photosynthetic proteins [17,34,40,41,42]. In another article from the current issue [34], we used it to determine how LHCII organization changes upon variations in pH and detergent concentration. The present paper stepped further, and we applied FCS to estimate more precise differences in the oligomerization state of an algal antenna protein from *C. velia* CLH. Additionally, we also estimated fluorescence intensities at the particle level. The method’s resolution was assessed by measuring preparations of LHCII monomers and trimers, as previously done by Janik and coworkers [17]. The results obtained were in line with classical biochemical methods (Figure 2) and confirmed that FCS is able to resolve between LHCII monomers and trimers (Figure 1).

CLH oligomerization state was recently an object of debate (e.g., [5,6,32]). Here, FCS data indicated that these antenna particles seem to be in the monomeric state in the condition we tested (Table 1, Figure 1 and Figure 2). We have to point out that particle size as detected in FCS does not translate directly into molecular weight values. Indeed, the estimated radius of LHCIIm was around half that of LHCIIt, whilst the molecular weight of the monomer is considered one-third the trimer (Table 1 and Table 3). This fact can be explained considering that FCS does not measure a “pure protein” size, but rather a radius comprising the actual protein size increased by lipids, detergent, and the overall hydrodynamic volume. It is important to note that these last parameters do not change proportionally with the oligomerization state of proteins (see e.g., [17,34]). Similarly, the changes in fluorescence emissions between LHCII monomers and trimers (or CLH) were not directly proportional to the increase in chlorophyll concentration in the particle (Table 2). These results altogether indicate that antennas represent a complex system, whose behavior is not just a simple sum of its components, pigments, and proteins. An antenna oligomer can then be considered as a synergic system of higher level compared to its separated subunits.

Sucrose gradients confirmed a monomeric state of the antenna from *C. velia* (Figure 2) in line with FCS data (Table 1, Figure 1). We want to note that this conclusion is valid for our experimental conditions, in particular growth conditions and antenna protein isolation protocol. Therefore, our data is not necessarily in contradiction with previous reports showing a multimeric state of CLH (e.g., [5,6,32]). It has been already pointed out that the oligomerization pattern can be affected by a different protein isolation technique [43] or by growth light intensity [16,17]. Indeed, it was already shown that the light conditions change the protein composition of CLH antennas [6]. The question of the oligomerization state of CLH at different conditions is thus still an open question that requires high-resolution structural data. Finally, the trimeric state previously assigned to CLH by Tichý and coworkers in similar solubilization conditions was based on electron microscopy maps of CLH after negative staining [32]. These authors compared the observed sizes of CLH antenna with antenna complexes from diatoms (FCP complex) and trimeric plant LHCII (see discussion in [32]). Due to technical limitations, however, negative staining in electron microscopy does not allow high-resolution structures for these types of small antenna proteins [44]. Moreover, recent publications revealed that the oligomeric state of other algal diatom antennas, e.g., FCP in diatoms, is largely diversified and species-dependent [31]. Indeed, high-resolution structures of tetrameric [10,11], trimeric [12], dimeric [30], and also monomeric [10,11,12] organization have been reported for diatom antennas alone. All these results show that a more general picture of the algal antenna oligomerization requires more systematic and comparative research.

We want to note, however, that comparative studies with antennas from different species need to also consider the different molecular weights of antennas from different species. Indeed, the antenna proteins of many red-clade algae are smaller than the LHC proteins of the green line. In fact, this size difference, multiplied by the number of monomers in multimeric antennas, can lead to large errors. In Table 3, we present an example of this, showing that the plant LHCII trimer is actually closer in size to a CLH *tetramer*. This is possibly the case, for instance, for the 8-nm particle described by Tichy and coworkers in their electron microscopy results [32].

Apart from their sizes, FCS data also allowed us to investigate the fluorescence properties of isolated antennas particles (Table 2). Interestingly, we observed a similarity between CLH and LHCIIm fluorescence when normalized to the number of chlorophylls (Table 2). The fluorescence yield of CLH was remarkably close to that of LHCIIm, both being higher than that of LHCIIt (Table 2). This result may indicate that protein oligomerization could affect some of the intrinsic fluorescence properties of the antennas. LHCII monomers usually display shorter fluorescence lifetimes compared to the LHCII trimers [14,45] meaning that monomers should be more quenched (i.e., with lower fluorescence) in comparison to trimers. The same effect one should expect also for fluorescence (count of photons) per protein. Further investigations will be necessary to explore and explain these points.

The monomeric LHCIIm and CLH particles have lower fluorescence (considering the CPP parameter) than the trimeric particles (Table 2). It is interesting to view our results in the light of pH-induced energetic quenching (qE), which is measured based on fluorescence quenching and is considered to be a photoprotective mechanism localized in the antennas. Our data are in line with previous results showing that qE is more efficient in monomeric antennas than LHCIIt [14,15,45]. Similarly, purified CLH antennas were found to be more susceptible to protonation and prone to dissipate light energy than LHCII trimers [35]. Interestingly, we could see that CLH fluorescence (measured as count per protein—CPP Table 2) is even smaller than CPP of the monomeric LHCIIm. It is worth mentioning that the different extent of quenching we observed between monomers and trimers could be also caused by their different sensitivity to pH. Trimeric antennas, indeed, are more protected from the environment than monomers, as they have residues hidden at the protein–protein interface. This could make them less susceptible to protonation, especially during in vitro experiments. Indeed, such effects were visible in the stronger response of monomeric LHC to solvent acidification than it is detectable in LHCII trimers [15,35]. However, we have to note that such effects might also be largely due to the nature of the proteins themselves. Indeed, most studies on monomeric antennas were performed on purified Lhcb4, Lhcb5, and Lhcb6 proteins, with slight differences in behavior between them (see e.g., [15]).

Such a difference in sensitivity to the environment, though, might be linked to the high diversity of structural organizations displayed by algal antennas. In a water column, the environmental conditions are susceptible to display large and fast changes [46]. Therefore, monomeric antennas, or particles with a high plasticity, might provide a faster and more efficient response than the stable oligomers forming the major antenna in the green line. Indeed, those are stabilized by a conserved “trimerization motif”, seemingly absent from the other clades [47]. It is worth reminding that most antenna proteins from eucaryotic phototrophs belong to the LHC superfamily [47] and thus present high sequence and structural similarity, which might explain in part their similar fluorescence behavior. We are thus tempted to suggest that, rather than major changes in the pigmentation and energetic pathways, changes in antenna oligomerization and plasticity were privileged across evolution to provide fast and efficient acclimation to various—and varying—environments.

These relations between protein, oligomeric state, and fluorescence quenching behavior will thus have to be further explored in future studies to be fully unraveled.

## 4. Materials and Methods

### 4.1. Antenna Protein Purification and Preparation

Plant thylakoid membranes were prepared from commercial spinach (*Spinacia oleracea*) leaves and trimeric LHCII light-harvesting antennas purified as described previously [34]. Monomerized LHCII was prepared from trimeric LHCII according to [48] with slight modifications. Before monomerization, the remaining sucrose in the LHCII trimers samples was washed by successive cycles of concentration and dilution in 10 mM HEPES KOH, pH 7.3, 200 μM DDM in Amicon centrifugal filter units (Millipore, Merck, NJ, USA) with a molecular weight cut-off of 10,000. The washed sample was then incubated for 24 h at room temperature with agitation at a concentration of 300 μg of chlorophyll/mL in a buffer containing 10 mM HEPES KOH pH 7.3, 0.01% DDM, 20 mM CaCl_2_, and 10 μg/mL of phospholipase A2 from honey bee venom (Sigma Aldrich, St. Louis, MO, USA). After the treatment, the monomerized LHCII was then separated on sucrose gradients (Supplementary Figure S2) as described above and compared with solubilized thylakoids used as a control.

Algal CLH proteins were isolated from *Chromera velia* (strain RM12, grown at 200 μmol photons m^−2^ s^−1^, continuous light) based on a published protocol [35] with modifications in the gradient separation step. Briefly, the isolated membranes were solubilized by incubation at 1 mg/mL in 25 mM HEPES KOH pH 7.8 and 2% n-dodecyl-β-D-maltoside (DDM), for 1 h on ice in the dark. The solubilized membranes were then deposited on sucrose gradients made by freezing and thawing a solution of 10 mM HEPES KOH pH 7.3, 0.6 M sucrose, and 0.04% DDM. The gradients were then ultracentrifuged for 20 h at 4 °C and 40,000 rpm in an SW40 rotor (Beckman Coulter, Brea, VA, USA). The antenna protein band purified from these gradients, better resolved than with the previous protocol, displayed the same absorption characteristics as the CLH described to this day (see Supplementary Figure S3 and [32,49]).

### 4.2. Fluorescence Correlation Spectroscopy

Fluorescence Correlation Spectroscopy (FCS) was performed on a Zeiss LSM 880 microscope (Carl Zeiss Microscopy GmbH, Jena, Germany) equipped with C-Apochromat water 40×/1.2 objective. The confocal pinhole was adjusted to 45 μm, and protein samples were excited by HeNe laser (633 nm, 1.0 μW). Antenna fluorescence was detected by a GaAsP detector (642–695 nm, photon counting mode) as described in [34]. Before all FCS measurements, the isolated antenna solution was first washed with a 1% Bovine Serum Albumin (Sigma Aldrich, USA) solution. LHCII trimers, monomers, and CLH antennas were all measured at about OD676 = 0.015, in a solution of 10 mM HEPES KOH pH 7.3 and 200 μM (0.01%) DDM. For each antenna type, the experiment was repeated 7 times. Autocorrelation traces were collected at room temperature 10 min after antenna sample dilution. The FCS curves were fitted in the Zen Black (Carl Zeiss Microscopy GmbH, Jena, Germany) by a single diffusion component using the conventional Levenberg-Marquardt multi-tau algorithm [50]. Determination of the diffusion time, antenna particle radius, and their fluorescence parameters was performed as described previously [34].

### 4.3. Significance Level Statistics

FCS results from two data groups were compared by means of the Graphpad “*t*-Test calculator” tool. Data entries were: means, standard deviations, and N (number of FCS measurements per group); output was two-tailed *p*-values. Data was not considered significantly different for *p*-value > 0.05, significantly different for *p* ≤ 0.05 and for *p* ≤ 0.01 very significantly different.

## 5. Conclusions

In conclusion, our data comparing oligomerization state and fluorescence intensity per protein (parameter CPP, Table 1) support an important role of oligomerization in the regulation between energy harvesting mode versus energy dissipation mode useful for photoprotection. Additionally, the combination of FCS and biochemical methods allowed us to identify CLH antennas from *C. velia* as monomeric complexes. Their fluorescence (counts per particle) was also low, similarly to LHCIIm compared to the trimeric (LHCIIt). The results altogether indicate that protein oligomerization affects antenna a switch between light harvesting and energy dissipation modes in the thylakoid membrane.

## Figures and Tables

**Table 1:** Diffusion times, inferred radius, and number of light-harvesting antenna particles. The parameters were calculated from ACFs obtained by FCS (see Figure 1). Data represent averages and standard deviations for 7 independent measurements.

Sample	Diffusion Time (μs)	Particle Radius (nm)	Number of Protein Particles
LHCIIt	745.6 ± 81.2	9.2 ± 1.5	25.1 ± 4.6
LHCIIm	490.9 ± 73.8	6.1 ± 1.9	38.3 ± 9.5
CLH	483.8 ± 81.7	6.0 ± 1.0	50.7 ± 11.7

**Table 2:** Fluorescence properties of the antennas as determined based on ACFs obtained by FCS. Parameters: Total Photon Count Rate (kHz); Count of photons per antenna particle and per second (Count per Particle, Hz), calculated as the total fluorescence count divided by the number of particles in Table 1; The proposed number of chlorophylls (Nb Chl) per antenna particle based on literature (see Introduction); Count of photons per chlorophyll per second (Hz). Results are the average and standard deviation of 7 independent measurements.

Sample	Total Photon Count Rate (kHz)	Count per Protein Particle (CPP) (Hz)	Nb Chl	Count per Chlorophyll (Hz)
LHCIIt	7.44 ± 1.34	299.57 ± 42.66	42	7.13 ± 1.02
LHCIIm	7.53 ± 0.69	207.86 ± 56.56	14	14.85 ± 4.04
CLH	6.50 ± 1.40	135.43 ± 45.32	8	16.93 ± 5.66

**Table 3:** Theoretical comparison of the sizes of LHCII and CLH oligomers. Calculation of the approximate molecular weight (MW) of different CLH oligomers, compared to LHCII monomers and trimers. For LHCII, we considered an averaged MW of 25 kDa for the apoprotein, as well as 14 Chl and 4 carotenoids per monomer, of an average MW of 900 and 600 Da, respectively. For CLH, the MW of the apoprotein presented here is the average of the 18 and 19 kDa proteins composing the purified antenna band [32]. We counted a theoretical 8 Chl and 4 Car per monomer, from bibliographic sources (see Introduction). Lipids were not considered, due to lack of information on CLH antennas.

Complex	Approximate MW Apoprotein (Da)	Nb Chl	Nb Car	Approximate Total MW (Da)
CLH monomer	18,500	8	4	28,100
**LHCII monomer**	**25,000**	**14**	**4**	**40,000**
CLH dimer	37,000	16	8	56,200
CLH trimer	55,500	24	12	84,300
CLH tetramer	74,000	32	16	112,400
**LHCII trimer**	**75,000**	**42**	**12**	**120,000**

## Data Availability

Not applicable.

## Supplementary Materials

The following are available online at https://www.mdpi.com/article/10.3390/ijms23020778/s1.
