# Supplementary material for: Size and Fluorescence Properties of Algal Photosynthetic Antenna Proteins Estimated by Microscopy
Source: Int J Mol Sci. 2022 Jan 11;23(2):778. doi: 10.3390/ijms23020778 (PMC8775774; doi:10.3390/ijms23020778)
Supplement: Supplementary file 1 [file ijms-23-00778-s001.zip › ijms-1537742-supplementary.pdf]

Supplementary Information

Size and fluorescence properties of algal photosynthetic antenna proteins estimated by microscopy

Aurélie Crepin[1,2], Erica Belgio[1], Barbora Šedivá[1], Eliška Kuthanová Trsková[1], Edel Cunill-Semanat[1], Radek Kaňa[1]

1 Centre Algatech, Institute of Microbiology of the Czech Academy of Sciences, Opatovický mlýn, 379 81 Třeboň, Czech Republic;
2 Current address: Umeå Plant Science Centre (UPSC), Department of Plant Physiology, Umeå University, 901 87, Umeå, Sweden;

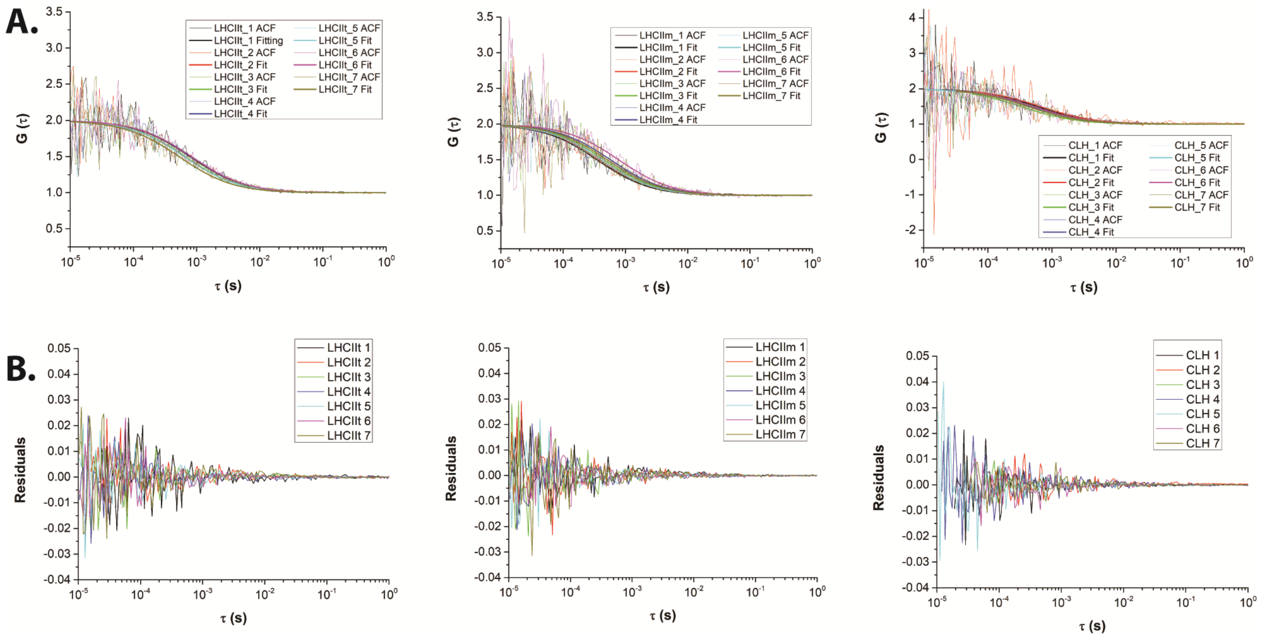

Supplementary Figure S1: Raw FCS results. A) Individual FCS traces and fittings. B) Residuals from the fittings in (A).

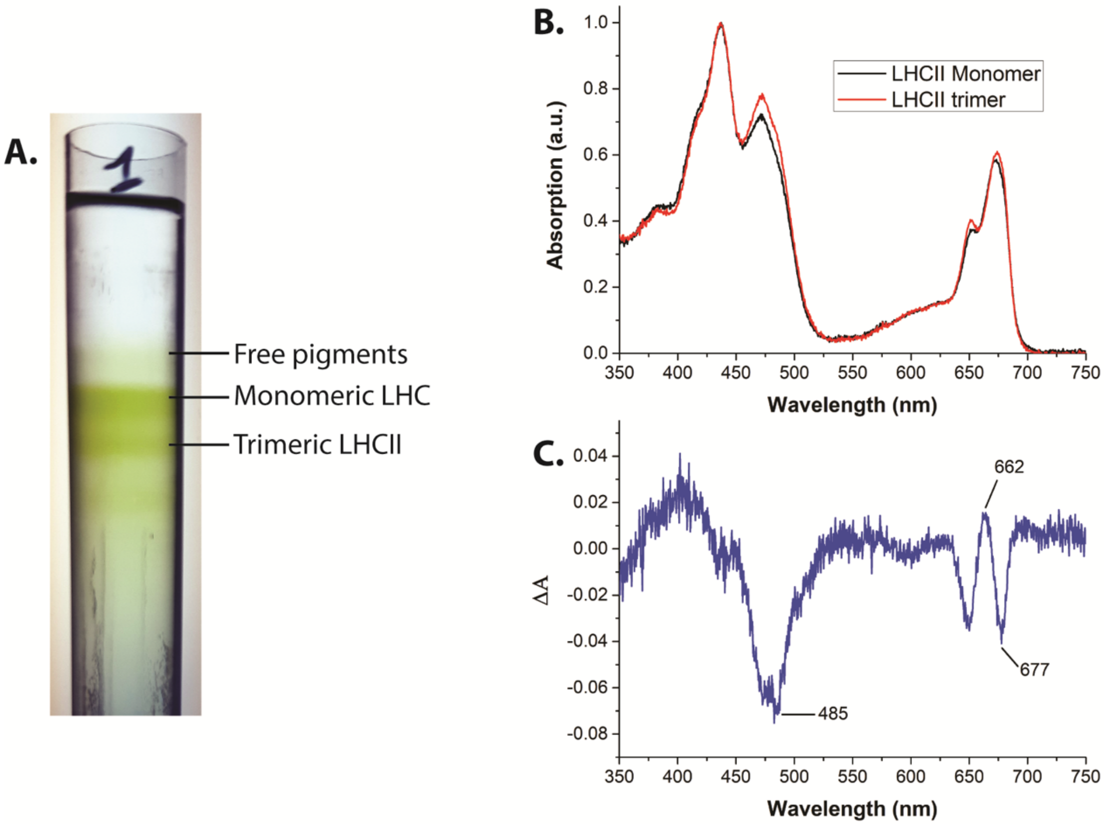

Supplementary Figure S2: LHCII monomerization. Purified LHCII trimers were treated with Phospholipase A2 to induce monomerization. A) Separation on sucrose gradient of the LHCII monomers and leftover trimers. B) Absorption spectra of the resulting LHCII monomers and trimers. C) Difference of the absorption spectra (calculated as Abs Monomer - Abs Trimer). The main peaks correspond to the ones typically observed upon LHCII monomerization, due to pigment rearrangement (see [1]).

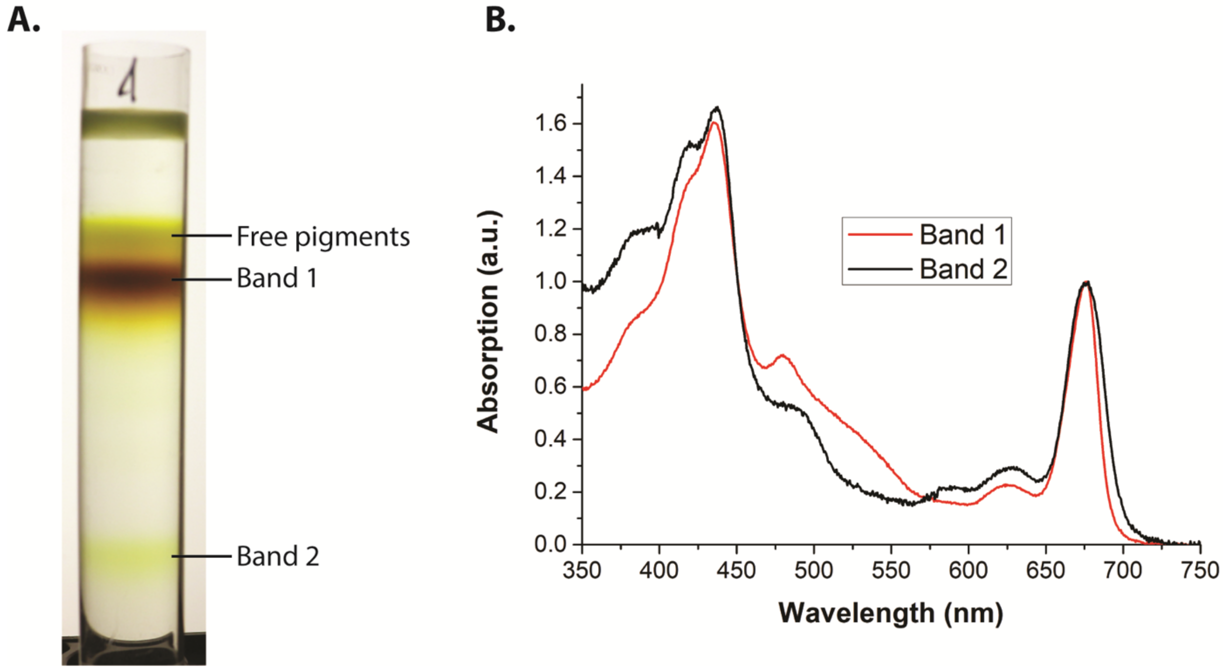

Supplementary Figure S3: CLH purification on sucrose gradients made by the freezing-thawing method. (A) Separation on sucrose gradients of solubilized membranes. Apart from the free pigments, two protein bands are visible. They were identified as CLH and PSI, respectively, based on absorption spectra and previous results (see e.g. [2-4]). (B) Absorption spectra of the bands purified from the gradient in (A).

References

[1] Wentworth, M., Ruban, A.V. and Horton, P. (2004). The functional significance of the monomeric and trimeric states of the photosystem II light harvesting complexes. Biochemistry 43, 501-9.

[2] Tichy, J., Gardian, Z., Bina, D., Konik, P., Litvin, R., Herbstova, M., Pain, A. and Vacha, F. (2013). Light harvesting complexes of Chromera velia, photosynthetic relative of apicomplexan parasites. Biochim Biophys Acta 1827, 723-9.

[3] Kana, R., Kotabova, E., Kopecna, J., Trskova, E., Belgio, E., Sobotka, R. and Ruban, A.V. (2016). Violaxanthin inhibits nonphotochemical quenching in light-harvesting antenna of Chromera velia. Febs Letters 590, 1076-1085.

[4] Kuthanova Trskova, E., Belgio, E., Yeates, A.M., Sobotka, R., Ruban, A.V. and Kana, R. (2018). Antenna proton sensitivity determines photosynthetic light harvesting strategy. Journal of Experimental Botany 69, 4483-4493.
